# A Nonhuman Primate Scrub Typhus Model: Protective Immune Responses Induced by pKarp47 DNA Vaccination in Cynomolgus Macaques

**DOI:** 10.4049/jimmunol.1402244

**Published:** 2015-01-19

**Authors:** Daniel H. Paris, Suchismita Chattopadhyay, Ju Jiang, Pruksa Nawtaisong, John S. Lee, Esterlina Tan, Eduardo Dela Cruz, Jasmin Burgos, Rodolfo Abalos, Stuart D. Blacksell, Eric Lombardini, Gareth D. Turner, Nicholas P. J. Day, Allen L. Richards

**Affiliations:** * Mahidol-Oxford Tropical Medicine Research Unit, Faculty of Tropical Medicine, Mahidol University, Bangkok 10400, Thailand;; † Centre for Tropical Medicine and Global Health, Nuffield Department of Clinical Medicine, University of Oxford, Oxford, OX3 7FZ, United Kingdom;; ‡ Viral and Rickettsial Diseases Department, Naval Medical Research Center, Silver Spring, MD 20910;; § Division of Entomology, Walter Reed Army Institute of Research, Silver Spring, MD 20910;; ¶ Leonard Wood Memorial Institute, Cebu 6000, Philippines;; ‖ Veterinary Medicine Department, Armed Forces Medical Research Institute of Science, Thanon Yothi, 10400 Bangkok; and; # Preventive Medicine and Biometrics Department, Uniformed Services University of the Health Sciences, Bethesda, MD 20814

## Abstract

We developed an intradermal (ID) challenge cynomolgus macaque (*Macaca fascicularis*) model of scrub typhus, the leading cause of treatable undifferentiated febrile illness in tropical Asia, caused by the obligate intracellular bacterium, *Orientia tsutsugamushi*. A well-characterized animal model is required for the development of clinically relevant diagnostic assays and evaluation of therapeutic agents and candidate vaccines. We investigated scrub typhus disease pathophysiology and evaluated two *O. tsutsugamushi* 47-kDa, Ag-based candidate vaccines, a DNA plasmid vaccine (pKarp47), and a virus-vectored vaccine (Kp47/47-Venezuelan equine encephalitis virus replicon particle) for safety, immunogenicity, and efficacy against homologous ID challenge with *O. tsutsugamushi* Karp. Control cynomolgus macaques developed fever, classic eschars, lymphadenopathy, bacteremia, altered liver function, increased WBC counts, pathogen-specific Ab (IgM and IgG), and cell-mediated immune responses. Vaccinated macaques receiving the DNA plasmid pKarp47 vaccine had significantly increased *O. tsutsugamushi*–specific, IFN-γ–producing PBMCs (*p* = 0.04), reduced eschar frequency and bacteremia duration (*p* ≤ 0.01), delayed bacteremia onset (*p* < 0.05), reduced circulating bacterial biomass (*p* = 0.01), and greater reduction of liver transaminase levels (*p* < 0.03) than controls. This study demonstrates a vaccine-induced immune response capable of conferring sterile immunity against high-dose homologous ID challenge of *O. tsutsugamushi* in a nonhuman primate model, and it provides insight into cell-mediated immune control of *O. tsutsugamushi* and dissemination dynamics, highlights the importance of bacteremia indices for evaluation of both natural and vaccine-induced immune responses, and importantly, to our knowledge, has determined the first phenotypic correlates of immune protection in scrub typhus. We conclude that this model is suitable for detailed investigations into vaccine-induced immune responses and correlates of immunity for scrub typhus.

## Introduction

Scrub typhus is caused by infection with *Orientia tsutsugamushi*, an obligate intracellular bacterium, and represents a major cause of undifferentiated febrile illness in a region covering 13,000,000 km^2^ in the Asia-Australia-Pacific region (1). Recent reports of severe disease, potential antibiotic resistance in southern India and northern Thailand, and increasing human case reports beyond endemic boundaries, for example, Africa, United Arab Emirates, and Chile, highlight the increasing clinical significance of this treatable infectious disease (2–7). Scrub typhus usually presents with nonspecific signs and symptoms but can rapidly lead to complications including pneumonia, renal failure, meningoencephalitis, vascular shock, gastrointestinal bleeding, myocarditis, and death (8–10). The current understanding of immunopathophysiological mechanisms associated with scrub typhus is limited by the lack of a well-characterized laboratory animal model for in-depth investigations of host responses during both the natural disease course and Ag-induced immune responses. Most of the animal models used in the past have been small animals inoculated/challenged via various routes including i.p. or i.v. Only recently has the intradermal (ID) route been evaluated, which, in contrast with the i.p. and i.v. routes, more closely resembles the mite feeding process and the natural route for *O. tsutsugamushi* infection in nature (11). Although these models allow for detailed immunological studies, they may not appropriately represent human scrub typhus and may therefore not be the best models to use for vaccination studies. Vaccines proven efficacious in these small-animal models have not been successful in humans (12–14). Thus, in addition to eliciting agent-specific immune responses and understanding their dynamics in the small-animal scrub typhus models, alternative models more closely aligned with human disease such as nonhuman primates (NHPs; macaques) should be considered.

Historically, cynomolgus, silvered leaf, and Indian rhesus macaques have been used as NHP models for scrub typhus because they display clinical signs and symptoms that are similar to those described for humans with scrub typhus (15–18). Cynomolgus macaques have been considered appropriate for studying human scrub typhus and have been recommended for use during the development of vaccines (14, 18). Cynomolgus macaques infected via ID needle inoculation with human-pathogenic *O. tsutsugamushi* (Karp strain) can develop scrub typhus–like eschars and/or dusky plaques within 7 d of inoculation, accompanied by inguinal lymphadenopathy and concomitant induction of anti–*O. tsutsugamushi*–specific IgM and IgG Abs (19, 20). Immunohistochemistry analysis of biopsies from eschars and enlarged regional lymph nodes demonstrated the presence of *O. tsutsugamushi*, and pathological features resembled human disease (20, 21). Previous immunogenicity and safety evaluations of a candidate vaccine against scrub typhus in cynomolgus macaques showed that a truncated, recombinant, 56-kDa outer membrane protein of the Karp strain of *O. tsutsugamushi* (Kp r56) was well tolerated and could induce strong humoral and cellular immune responses. Specifically, the recombinant protein induced strong Ag-specific IgM and IgG titers, and after stimulation, the PBMCs from the vaccinated animals showed an Ag-specific induction of proliferation and an increase in IFN-γ production (19). Because a well-characterized animal model is required for the development of clinically relevant therapeutic agents and vaccine candidates, we investigated the pathophysiological mechanisms of scrub typhus in cynomolgus macaques as a means to model human disease and to evaluate candidate vaccines. In this study, we used the highly conserved *O. tsutsugamushi* 47-kDa Ag (HtrA) gene as a vaccine candidate in two different vaccine formulations (22). The two different vaccines based on the 47-kDa protein were a pKarp47 DNA plasmid vaccine with the molecular adjuvant pGM-CSF and a virus-vectored vaccine Kp47/47-Venezuelan equine encephalitis virus (VEE) replicon particle (VRP). The former has been shown to stimulate a robust and long-lasting cellular immune response in CD-1 outbred mice (23). Moreover, the plasmid vaccine was able to provide complete protection to immunized mice against homologous challenge and, to a limited degree, to a heterologous challenge (23). Using these candidates, we evaluated the NHP vaccine responses in two homologous and one heterologous prime boost regimen, and documented the safety, immunogenicity, and protective efficacy against a subsequent homologous challenge with *O. tsutsugamushi* Karp.

## Materials and Methods

### Ethical approval

The animal protocol was approved by the Leonard Wood Memorial Institute’s Institutional Animal Care and Use Committee in accordance with provisions of the U.S. Department of Agriculture Animal Welfare Act, the Public Health Service Policy on Humane Care and Use of Laboratory Animals, and the U.S. Interagency Research Animal Committee Principles for the Utilization and Care of Research Animals.

### NHPs

Twenty captivity-reared male Philippine cynomolgus macaques (*Macaca fascicularis*) of ∼2–3 y of age and 3–4 kg weight, with normal physical examinations and routine clinical laboratory assessments, were assigned to the study. Serological screening revealed no previous exposure to *O. tsutsugamushi*, SIV, hepatitis B virus, *Herpesvirus simiae* (B virus), or filovirus, and all had a negative tuberculin test within the last 12 mo.

Two attending veterinarians performed all blood draws and skin biopsies after i.m. administration of a mixed sedative containing 5 ml tiletamine hydrochloride/zolazepam hydrochloride (50:50 at 16.7 mg/ml each), 1.5 ml xylazine hydrochloride (100 mg/ml), and 1.0 ml atropine sulfate (0.65 mg/ml), and applying a dosage of 0.1 ml/kg body weight. All animals survived the challenge phase of the study.

### *O. tsutsugamushi* Karp inoculum and challenge

The human-pathogenic *O. tsutsugamushi* Karp strain from Papua New Guinea (24) was propagated in CD1 Swiss mice via i.p. injection, and the murine LD_50_ (MuLD_50_) was determined as previously described (25). The inoculum was prepared using homogenized livers and spleens of moribund mice, diluted in Snyder’s I buffer, and all animals were challenged with 10^6^ MuLD_50_ as described previously (19, 25). ID inoculation via a 26-gauge needle and injection of 100 μl homogenate per site in the anteromedial aspect of the thigh was performed by a veterinarian in accordance with the Guide for the Care and Use of Laboratory Animals (26).

### Vaccines and vaccinations

#### Vaccines.

Two vaccine formulations were used, each as a homologous triple-boost regimen and one regimen as a combined heterologous prime-boost strategy.

##### Kp47/47-VRP.

This vaccine consisted of a VRP that encoded the Karp strain 47-kDa Ag. This vaccine vector is composed of self-replicating RNA containing all of the VEE nonstructural genes and *cis*-acting elements, and two copies of the whole open reading frame of the 47-kDa Ag gene including the secretory signal sequence, termed Kp47/47-VRP, cloned downstream of two independent subgenomic 26S promoters, that were inserted in place of the viral structural genes (27–29). Administration of this virus-vectored vaccine candidate consisted of 10^7^ focus-forming units (FFUs) of Kp47/47-VRP per milliliter PBS injected i.m. into the anterolateral aspect of the thigh.

##### pKarp47/pRhGM-CSF.

pKarp47 consisted of the entire open reading frame of the 47-kDa gene from the Karp strain of *O. tsutsugamushi*, and the molecular adjuvant pRhGM-CSF consisted of the Rhesus GM-CSF coding sequence, individually cloned into the eukaryotic expression vector pVR-1012 (Vical, San Diego, CA) (30). Coadministration of the vaccine candidate pKarp47 and the genetic adjuvant pRhGM-CSF in 1 ml PBS consisted of 2000, 1000, or 500 μg pKarp47, and 10% of that amount of pRhGM-CSF, that is, 200, 100, and 50 μg per macaque, respectively, injected i.m. into the anterolateral aspect of the thigh. The vaccine candidate pKarp47 was always coadministered with the molecular adjuvant pRh-GM-CSF; therefore, future reference to pKarp47 in this article refers to the candidate coadministered with its adjuvant.

#### Vaccine groups.

There were a total of four vaccine groups in this study: group 1 (control group, *n* = 6) received PBS (*n* = 2), 2000 μg pVR1012 (*n* = 1), 200 μg pRhGM-CSF (*n* = 2), and 10^7^ mutagenized staphylococcal enterotoxin B replicon particle (mSEB-VRP) (*n* = 1); group 2 (*n* = 2) received three doses of Kp47/47-VRP; group 3 (*n* = 6) received three doses of pKarp47 at three dosage levels; and group 4 (*n* = 6) received a heterologous prime-boost consisting of two consecutive pKarp47 vaccinations (analogous to group 3) followed by a boost with Kp47/47-VRP (analogous to group 2).

#### Vaccination schedule.

The four groups received their vaccinations on day 0, with booster vaccinations at 3-wk intervals. At the second booster vaccination, group 3 received a third dose of pKarp47 [termed pKarp47(×3)], and group 4 received a heterologous dose of Kp47/47-VRP (termed pKarp47(×2) + Kp47/47-VRP).

#### Challenge.

Three weeks after the final vaccination, all macaques were ID challenged with 10^6^ MuLD_50_ of the Karp strain of *O. tsutsugamushi* on the left thigh.

### Clinical observations

Clinical observations started 1 wk before initial vaccination and continued until the end of the study (i.e., 28 d after challenge). The animals were viewed twice daily for signs of morbidity, and full physical examinations were conducted every second day (including skin and fur characteristics, eye and mucous membranes, respiratory, circulatory, autonomic and CNSs, somatomotor, and behavioral patterns). Body temperatures (rectal) and food consumption were monitored before each inoculation and daily thereafter. Skin observations at both the vaccine inoculation site and at the challenge site were recorded every 2 d and scored following the Draize algorithm as described previously (see Table I footnote) (19, 20).

Fever was defined as >37°C, which corresponded to the mean of all baseline measurements of all macaques +2 SEs as previously described (26). Blood samples were collected via the saphenous vein before each vaccination and booster vaccination, and 3 wk after the final boost to conduct hematological and chemical assessments (Supplemental Material), IgG- and IgM-specific *O. tsutsugamushi* Ab level determinations (ELISA), and ELISpot assays for IFN-γ.

### Quantitation of bacteremia (*O. tsutsugamushi* 22-kDa protein gene quantitative real-time PCR assay)

A series of 0.5 ml EDTA blood samples was collected daily the first 3 d after challenge, then every second day during the study to determine the onset and duration of bacteremia by using a quantitative real-time PCR (qPCR) assay based on the *O. tsutsugamushi* 22-kDa protein gene (Otsu22). EDTA whole blood was stored at −80°C until assessed. Upon thawing, DNA was extracted from 200 μl blood using a QIAamp DNA Blood Mini Kit (Qiagen, Valencia, CA) following manufacturer’s instructions, and 100 μl elution buffer was used to elute the DNA. Five microliters of the DNA preparations was used in the Otsu22 qPCR assay.

The Otsu22 qPCR assay targeted the conserved region of the 22-kDa outer membrane protein gene based on the sequence alignment of eight *O. tsutsugamushi* strains (Karp, Kato, TA763, AFSC7, 18-032460, TH1814, MAK119, and Boryong) (31). The forward and reverse primers (Ot22-640F: 5′-TGCAGGMATAMAAACTGTTACTA-3′ and Ot22-784R: 5′-AGCTAATCCYTCTGCTCCTAAA-3′) amplified a 145-bp fragment of the Otsu22, which is recognized by a MolecularBeacon probe (Ot22-722BP: 5′-CGCGATCTGCTGCTTCTCCAGCTTCAATCGCG-3′) labeled with 5′-6FAM and BHQ-1-3′. Each qPCR was carried out using a SmartCycler II System (Cepheid, Sunnyvale, CA) and OmniMix reagents (Cepheid) with forward and reverse primers (0.6 μM each), probe (0.5 μM), and optimized MgCl_2_ (7 mM). All qPCRs were incubated at 94°C for 2 min followed by 50 cycles of two-step amplification at 94°C for 5 s and 58.5°C for 30 s. Under the optimized conditions, this assay was found to be sensitive with a limit of detection of three DNA copies per reaction as determined by a dilution series of DNA plasmids as external standards (*r*^2^ value of the standard curve was 0.999 with 99.1% efficiency). This assay is species specific; it recognized 21/22 isolates of *O. tsutsugamushi*, including Karp, but not *O. tsutsugamushi* Brown, rickettsial (*n* = 6), or nonrickettsia bacterial (*n* = 11) DNA evaluated. It also did not react with human, mouse, or monkey genomic DNA. The following strains were included: *O. tsutsugamushi*: Karp, Kato, Gilliam, TA763, TH1811, TH1812, TH1826, AFC-1, AFC-3, AFC-30, AFPL-12, MAK-110, MAK-119, MAK-243, R-11666, 18-032460, Brown, Citrano, Domrow, Garton, Woods, and Ikeda; *Rickettsia*: *R. akari* VR612, *R. felis* Cal2, *R. parkeri* C, *R. rickettsii* VR891, *R. prowazekii* Breinl, and *R. typhi* Wilmington; and nonrickettsial species: *Salmonella enterica, Proteus mirabilis, Escherichia coli, Corynebacterium* spp., *Legionella micdadei* Tatlock, *Bartonella vinsonii, Bartonella quintana, Neorickettsia risticii, Neorickettsia sennetsu, Francisella persica,* and *Staphylococcus aureus*.

### Eschar biopsies

Skin biopsies were obtained postchallenge from two macaques at day 2 (ID8749 and ID5253) and day 7 (ID6856 and ID5234). Biopsies were performed with disposable 8-mm circular punch biopsies (Stiefel Laboratories, Offenbach, Germany) to simultaneously sample the inoculation site (central necrosis and perifocal inflamed skin) and a distant site of unaffected skin as control. Each biopsy was sectioned into four equal quarters, separately fixed in 4% glutaraldehyde (for electron microscopy), OCT (optimal cryosectioning compound, for frozen sectioning), PBS (for PCR), or 10% neutral-buffered formalin (processed later into paraffin blocks, for immunohistochemistry) (21).

### Samples for biochemistry, hematology, and immunology

All macaques were bled via saphenous veins before challenge and afterward at days 7, 14, 21, and 28 for chemical, hematological, and immunological analyses (raw data in Supplemental Material). Blood chemistry analyses included protein, albumin, calcium, phosphorus, creatinine, aspartate aminotransferase, alanine aminotransferase, globulin, alkaline phosphatase, cholesterol, triglycerides, total bilirubin, urea nitrogen, carbon dioxide, albumin/globulin ratio, glucose, and creatinine kinase, and were performed on serum separated from clotted blood (1.5 ml blood). Hematological analyses included complete blood count, RBC count, hemoglobin, hematocrit, platelet count leukocyte count, leukocyte differential, mean RBC volume, mean RBC hemoglobin, mean RBC hemoglobin concentration, and mean platelet volume, and were performed using EDTA-anticoagulated blood (1.0 ml). Cellular immunological studies used PBMCs separated from 4 ml EDTA-anticoagulated blood using Ficoll-Hypaque gradients (Pharmacia, Peapack, NJ). Plasma was collected and stored at −80°C for use in Ab studies. The Ficoll-Hypaque gradient cell layers were transferred to test tubes, washed twice with PBS, resuspended in freezing medium (containing 10% DMSO in FBS [Invitrogen, Carlsbad, CA]), and frozen at ≤−80°C until assessed using Ag-specific ELISpot assays.

### Safety of vaccines

A large panel of biochemical and hematological markers (*n* = 26) was measured at various time points throughout the study: baseline, three time points per vaccination (triple regimens) and during challenge (days 7, 14, 21, and 28), leading to a total of 14 time points per individual marker. These markers were then used to correlate any abnormal biochemical or hematological reactions associated with the vaccines or the vaccination process.

### Adaptive immune response assays

Macaques were bled once before infection and at weekly intervals thereafter, to assess their immune responses to infection.

#### Humoral response.

Plasma samples were thawed and tested for the presence of Ag-specific Abs using ELISAs for the detection of IgM and IgG. The ELISA assays used a recombinant protein, 47-kDa Ag strain Karp (Kp r47b), supplied by Invitrogen (0.05 μg/well; Carlsbad, CA), or *O. tsutsugamushi* Karp whole-cell Ag (at 0.1 μg/well) as previously described (23, 32). Isotyping was performed using recombinant protein Kpr47b as the Ag detected with HRP-conjugated anti-human IgG1, IgG2, IgG3, and IgG4 (Serotec, Raleigh, NC) as described previously (33).

#### Cell-mediated response.

PBMCs were thawed and stimulated ex vivo with 1.0 μg/well Kp r47b for 36 h to assess the cellular immune response to *O. tsutsugamushi* infection. ELISpot assays were used to detect and quantify cells induced to secrete IFN-γ and IL-13 (data not shown) as previously described using the CTL ImmunoSpot Analyzer (Cellular Technology, Cleveland, OH) (23).

### Necropsy procedures

Upon completion of the challenge study period (30 d), full autopsies were performed and tissue samples were taken from all major organs according to a standardized protocol. Tissue samples for bacterial load quantitation were digested using protease K, and DNAs were extracted and bacterial loads determined using the Otsu22 qPCR assay. Ratios comparing a cynomolgus housekeeping gene (oncostatin M) qPCR assay results with those of the Otsu22 qPCR results were calculated to determine the bacterial density associated with tissue cell numbers (34). Tissues sample from all organs were fixed in 10% neutral-buffered formalin and processed into paraffin wax blocks for histopathological (H&E), immunoenzymatic (DAKO EnVisionTM+ System, HRP kit; DAKO, Glostrup, Denmark), and immunofluorescent (double immunolabeling with a specific anti–*O. tsutsugamushi* Ab) analysis as previously described (21).

### Statistical analysis

Statistical calculations were performed with Stata/SE version 11 (College Station, TX) and Prism Software, version 6.0b (2012) for graphs. Comparisons between control and vaccine groups were performed using nonparametric tests (Wilcoxon rank sum test and Kruskal–Wallis test). Quantitation of bacteremia was performed by transforming the bacterial loads over time into total area under the curve (AUC) using Stata/SE software. Survival analysis included estimation of incidence densities and Kaplan–Meier survival curves, and comparison of survival function included the log-rank and Cox proportional hazards model. For investigation of vaccine efficacy and safety, the data were analyzed for differences between groups using the Kruskal–Wallis test, and for more detailed pairwise comparisons the rank sum test and logistic regression. The biochemical and hematological values were related to baseline values (baseline values served as covariates) using analysis of covariance (ANCOVA). All hypothesis testing was two-tailed, and a *p* value <0.05 was considered statistically significant. Statistical analyses did not include adjustments for multiple comparisons, resulting in more stringent overcalling of statistical associations, which benefited the safety analysis.

## Results

### Pathophysiology of scrub typhus

#### Inoculation site, lymphadenopathy, and fever.

**FIGURE 1.**
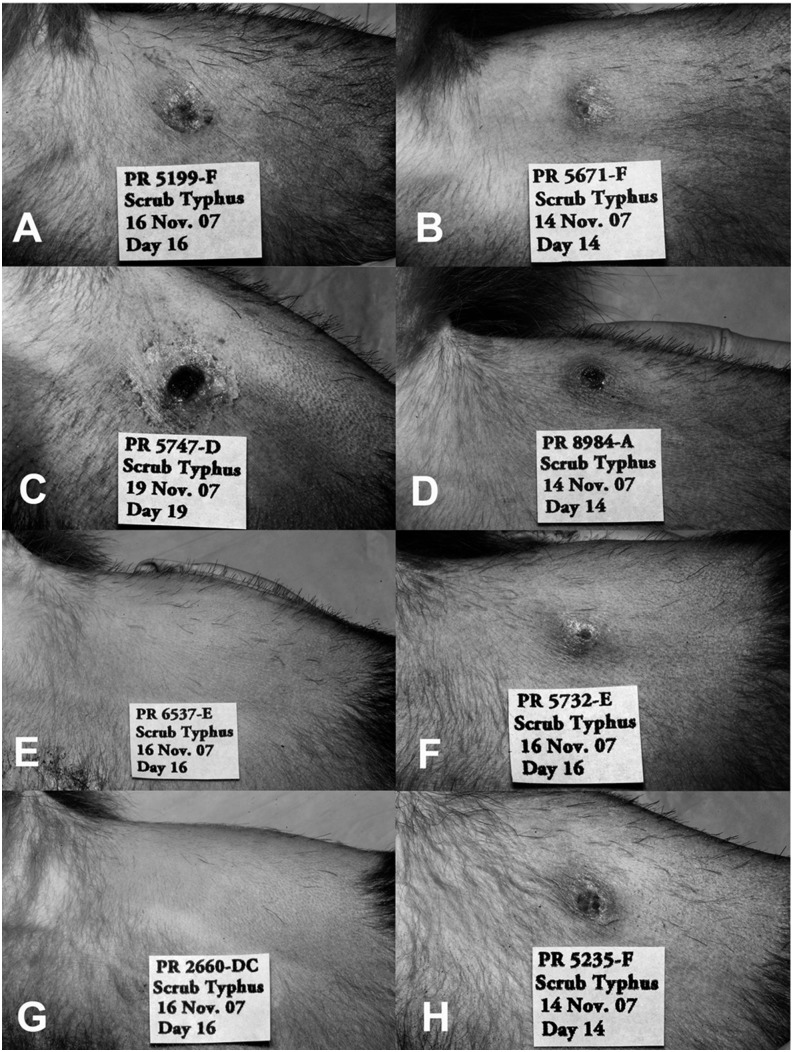
Cynomolgus macaques develop eschars at the ID inoculation site of *O. tsutsugamushi* Karp strain. Eschars developed at the ID inoculation site of *O. tsutsugamushi* Karp strain, on the left inner thigh in cynomolgus macaques. (**A**–**D**, **F**, and **H**) The eschar images represent the most prominent eschars observed in each group, that is, with the highest eschar scoring value, based on induration, ulceration, and inflammation (between days 14 and 19 after inoculation), or the lowest score achieved as a result of vaccination-associated immune response (**E** and **G**). Two representative images are shown per vaccine group. (A and B) Pictures of macaques in the control group (group 1, *n* = 6). (C) and (D) show macaques inoculated with the Kp47/47-VRP vaccine (group 2, *n* = 2), (E) and (F) received the pKarp47 vaccine as a triple-homologous prime-boost regimen (group 3, *n* = 6), and (G) and (H) received pKarp47 vaccine twice and a third boost with the Kp47/47-VRP vaccine candidate, as a heterologous prime-boost regimen (group 4, *n* = 6).

**FIGURE 2.**
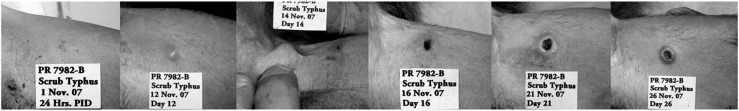
Time-course development of eschar and lymphadenopathy. Development of a bona-fide eschar after ID inoculation in a cynomolgus macaque (NHP ID7982). After ID inoculation of *O. tsutsugamushi* (10^6^ × MuLD_50_ of mouse liver-spleen homogenate), a papular lesion appeared after 12 d, regional lymph node enlargement at 14 d, central ulceration of an indurated lesion at 16 d, systemic lymph-node enlargement at 19 d, with regression of local inflammation and healing of eschar at 26 d, general lymphadenopathy could be observed until the end of the study at day 32.

All macaques challenged ID with *O. tsutsugamushi* developed eschars characterized by papular lesions with perifocal inflammation and induration starting 3–7 d after inoculation, and central excavation and crust formation between days 14 and 19 (Figs. 1 and 2).

All control macaques (*n* = 6) developed inguinal lymphadenopathy ipsilateral to the inoculation site followed by generalized lymphadenopathy. Lymph-node enlargement was observed at day 5, when 15/20 (75%) macaques developed regional lymphadenopathy, which increased to 100% by day 9 (Table I). The median time (range) to development of regional lymphadenopathy was 5 d (5–7) and for generalized lymphadenopathy was 13 d (7–16).

**Table I.** Overview of cynomolgus macaques (NHPs), vaccine groups, and dermal inoculation-site observations

				Draize Scores							
				Edema*^a^*		Erythema*^b^*		Eschar		Lymphadenopathy	
Vaccine Group	Vaccine	NHP ID	Inoc. No.	Max. Score	Days	Max. Score	Days	Max. Score	Days	Regional	General
Group 1a	None (PBS)	8749	9	B		B		B	D2	5	16
	None (PBS)	6741	3	3	12–14	3	12–16	3	12–19	5	7
Group 1b	None (pVR1012)	5671	8	3	12–14	3	9–14	3	12–16	5	9
Group 1c	None (pGM-CSF)	8824	12	3	12–14	3	12–14	3	9–14	7	16
	None (pGM-CSF)	5199	6	3	12–14	4	14–16	3	12–19	5	16
Group 1d	None (mSEB-VRP)	6856	18	B		B		B	D7	5	9
Group 2	Kp47/47-VRP	5747	4	3	9–16	4	9–14	4	9–16	5	16
	Kp47/47-VRP	8984	10	3	9–14	4	9–14	4	12–14	5	7
Group 3.1	pKarp47 (2 mg ×3)	6537	13	0	—	0	—	NE	—	5	16
	pKarp47 (2 mg ×3)	5732	1	3	12–16	3	14–16	3	16–21	5	16
Group 3.2	pKarp47 (1 mg ×3)	5253	19	B		B		B	D2	7	7
	pKarp47 (1 mg ×3)	7416	7	3	16–19	3	16–19	NE	—	9	16
Group 3.3	pKarp47 (0.5 mg ×3)	5817	15	0	—	0	—	NE	—	7	28
	pKarp47 (0.5 mg ×3)	5601	16	0	—	0	—	NE	—	7	14
Group 4.1	pKarp47 (2 mg ×2+VRP)	5689	2	2	12–14	2	12–14	NE	—	5	9
	pKarp47 (2 mg ×2+VRP)	6985	14	3	16–19	3	16–19	3	16–23	5	7
Group 4.2	pKarp47 (1 mg ×2+Kp47/47-VRP)	2660	20	0	—	0	—	NE	—	5	19
	pKarp47 (1 mg ×2+Kp47/47-VRP)	5234	5	B		B		B	D7	5	9
Group 4.3	pKarp47 (0.5 mg ×2+Kp47/47-VRP)	5235	11	2	14–16	3	12–14	3	12–19	5	12
	pKarp47 (0.5 mg ×2+Kp47/47VRP)	7982	17	3	16–19	4	16–19	4	16–26	5	12

Highest scores for edema, erythema, and eschar are noted and time period they were observed in days postinoculation. Lymphadenopathy denotes the first day of clinical enlargement of regional (draining) lymph nodes and generalized lymph nodes.

^*a*^ Edema scores: 0, none (no swelling); 1, minimal (slight swelling); 2, mild (defined swelling [distinct]); 3, moderate (defined swelling [raised]). Erythema scores: 0, none (normal color); 1, minimal (light pink); 2, mild (bright pink/pale red); 3, moderate (bright red); 4, severe (dark red).

^*b*^ Eschar scores: 1, papule (induration and inflammation); 2, excoriation (induration and central lesion); 3, central necrosis (central lesion with raised induration around the lesion, <10 mm); 4, central necrosis and raised induration (large central ulceration and black crust, >10 mm).

B, biopsy; Inoc., inoculation; Max. maximum; NE, no eschar.

**FIGURE 3.**
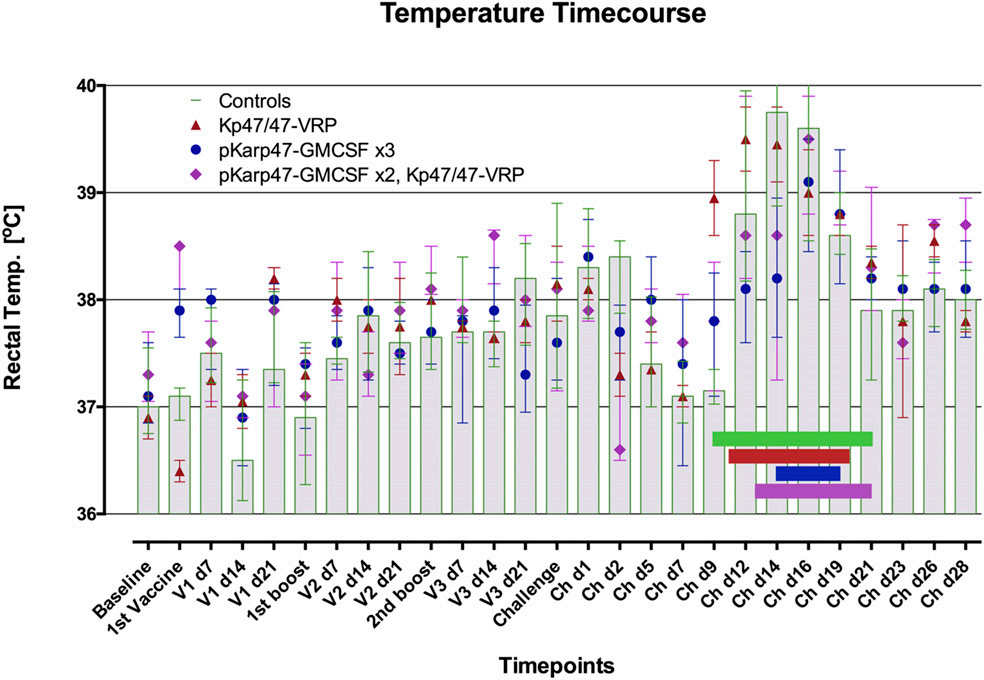
Rectal temperature progression caused by scrub typhus disease is affected by the different vaccine regimens administered. Macaques in the four vaccine groups demonstrated differences in onset, extent, and duration of fever, as well as bacteremia. Comparisons of AUC data for temperature and bacteremia showed close correlation (*p* = 0.034, Spearman’s rank correlation). The vaccinated macaques that achieved sterile immunity (2/5 in group 3 and 1/5 in group 4) did not develop febrile temperatures and eschars. Data are median (IQR) rectal temperature in degrees Celsius per vaccine group. Horizontal bars represent the median onset and duration of bacteremia, as defined by qPCR positivity in blood. NHPs with eschar biopsies were not included for this analysis. Control macaques (group 1, *n* = 6) are represented in green and gray columns; vaccinees with regimens Kp47/47-VRP in group 2 (*n* = 2), pKarp47 (×3) in group 3 (*n* = 6), and pKarp47 (×2) plus Kp47/47-VRP in group 4 (*n* = 6) are red, blue, and purple, respectively.

All control macaques developed fever during the bacteremic period (Fig. 3). The median (interquartile range [IQR]) temperature decline over the first 3 d after ID challenge with *O. tsutsugamushi* was −1.25°C (−1.4 to −0.5°C), followed by an increase of 2.1°C (1.7–2.9°C), and peaking at a median of 39.5°C (38.7–40.1°C) on day 14. No changes in weight or behavior patterns were observed in any study macaques.

#### Chemistry, hematology, and bacteremia.

Hematological and biochemical analyses of sera collected before and post ID infection of control macaques showed significant differences in levels of platelets, leukocyte and lymphocyte counts, protein, albumin, globulin, albumin/globulin ratio, alkaline phosphatase, urea nitrogen, and creatinine kinase (all *p* < 0.05).

Blood samples were quantitated for *O. tsutsugamushi* DNA copy numbers three times a week using the Otsu22 qPCR assay (Table II). All control macaques displayed bacteremia, with median (range) onset at 10.5 d (7–23) postinfection and a median duration of 12 d (10–15). Bacteremia peaked at day 16 postinfection in all control animals (Table II).

**Table II.** Effect of vaccination on bacteremia progression in NHPs inoculated ID with *O. tsutsugamushi* Karp

	Bacteremia*^a^*															
Vaccine Group	NHP ID	d5	d7	d9	d12	d14	d16	d19	d21	d23	d26	d28	Duration of Bacteremia (days)	Start of Bacteremia (days postinoculation)	AUC of Bacteremia*^b^*	Eschar Y/N/B*^c^*
Group 1																
None (PBS)	8749*^c^*				1	4	8	3					8	12	33.5	B (d2)
None (PBS)	6741		3	4	10	67	103	4					13	7	435.5	Y
None (pVR1012)	5671			2	7	14	43	1					11	9	157.5	Y
None (pGM-CSF)	8824				1	12	40	6	1				10	12	141	Y
None (pGM-CSF)	5199			2	11	65	570	91	14	4			15	9	1845	Y
None (mSEB-VRP)	6856*^c^*				7	18	25	6	4				10	12	124.5	B (d7)
Group 2																
Kp47/47-VRP	5747			3	18	119	551	188	13				13	9	2148	Y
Kp47/47-VRP	8984				7	8	38						5	12	61	Y
Group 3																
pKarp47 (2 mg ×3)	6537												0		0	**N**
pKarp47 (2 mg ×3)	5732					7	4	3					6	14	21.5	Y
pKarp47 (1 mg ×3)	5253*^c^*												0		0	B (d2)
pKarp47 (1 mg ×3)	7416				1	8	10	14					8	12	63	**N**
pKarp47 (0.5 mg ×3)	5817												0		0	**N**
pKarp47 (0.5 mg ×3)	5601							13	5	5			5	19	28	**N**
Group 4																
pKarp47 (2 mg ×2) +Kp47/47-VRP	5689					9	45	7	3				8	14	142	**N**
pKarp47 (2 mg ×2) +Kp47/47-VRP	6985					3	2	0	1				8	14	9	Y
pKarp47 (1 mg ×2) +Kp47/47VRP)	2660												0		0	**N**
pKarp47 (1 mg ×2) +Kp47/47-VRP	5234*^c^*			3	21	32	54	3	2				13	9	265.5	B (d7)
pKarp47 (0.5 mg ×2) +Kp47/47-VRP	5235				10	48	110	19	10	1			12	12	449.5	Y
pKarp47 (0.5 mg ×2) +Kp47/47-VRP	7982				4	6	13	23	81	9			12	12	277	Y

All unvaccinated NHPs in the control group developed bacteremia after ID challenge, whereas the period, start point, and AUC of bacteremia differed between the vaccine groups.

^*a*^ Bacteremia was measured as DNA GEs/10 μl whole blood.

^*b*^ AUC corresponds to the total sum of bacterial copy numbers during bacteremia as surrogate for the bacterial load during bacteremic dissemination phase.

^*c*^ Biopsy taken at the site of inoculation (day 2 or 7).

B, biopsy; d, day; ID, identification; N, no; Y, yes.

The macaques that had an eschar biopsy taken (*n* = 2) were excluded from these analyses, because this procedure can reduce the initial/subsequent bacterial loads. The macaque ID8749, biopsied at day 2, had the shortest duration of bacteremia (*n* = 8 d), the lowest median rickettsial DNA copies (3.5 genomic equivalents [GEs] per 10 μl full blood), and lowest peak bacterial load (8 GEs per 10 μl whole blood) compared with the other six control macaques (Table II).

#### Eschar biopsies.

**FIGURE 4.**
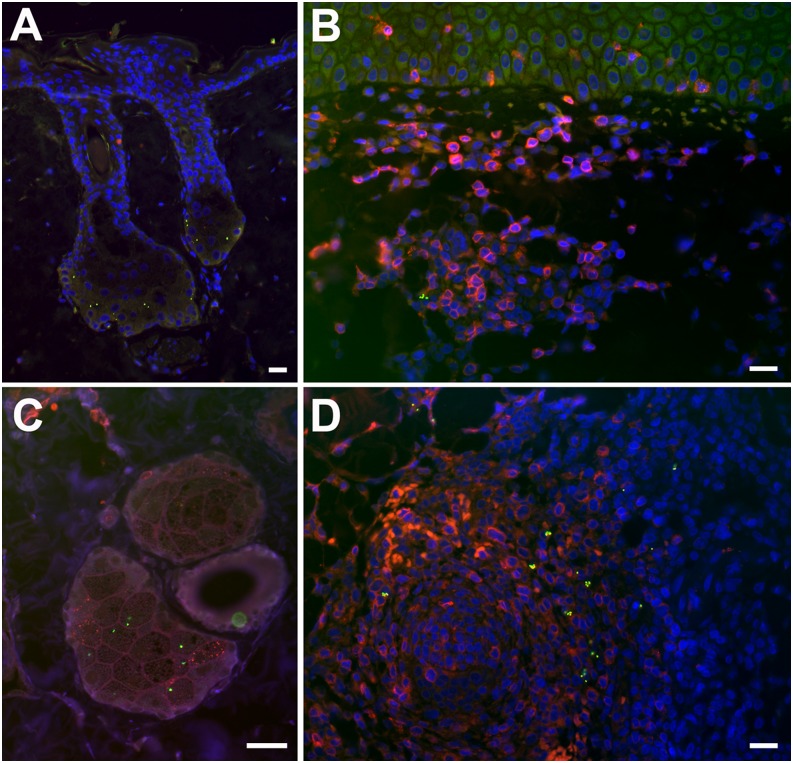
Early subepidermal infiltrates in eschar biopsies of cynomolgus macaques. Post-ID inoculation *O. tsutsugamushi* localize to the hair follicles and sebaceous glands (**A** and **C**) accompanied by formation of early CD3^+^ lymphocyte infiltrates. The subdermal infiltrates in cynomolgus macaques at day 7 postinoculation of *O. tsutsugamushi* are characterized by a large proportion of T lymphocytes (**B**), as well as formation of lymphocyte follicles in the deeper dermis (**D**), with localization of *O. tsutsugamushi* at the peripheral border, similar to the location also seen in spleen follicles (data not shown). Double immunolabeling with CD3 staining is in red and *O. tsutsugamushi* is in green. (A) NHP ID6856, day 2 biopsy. (B and D) NHP ID5234, day 7 biopsy. (**C**) NHP ID5234, day 2 biopsy. All images were taken using a Nikon Eclipse E400 microscope and Nikon digital camera (DS-L1; Nikon, Tokyo, Japan); images were merged and optimized with Photoshop CS3 extended, version 10.0. Original magnification ×100 (A); ×400 (B and D); ×600 (C). Scale bars, 20 μm.

**FIGURE 5.**
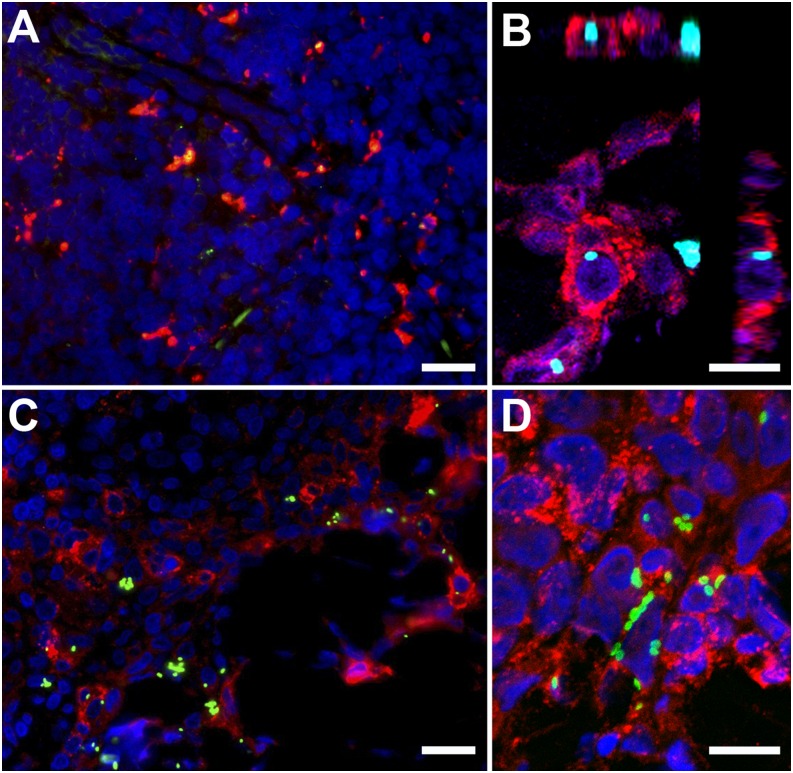
Eschars of *O. tsutsugamushi*–infected cynomolgus macaques at 7 d postinfection show bacteria colocalization with APCs. Skin eschar biopsies taken at 7 d after ID inoculation of *O. tsutsugamushi* into the anterior thigh of cynomolgus macaques demonstrate colocalization of *O. tsutsugamushi* predominantly with APCs. Internalized bacteria are depicted within macrophages (**A**) (CD68), within APCs (**B** and **C**) (HLA-DR), predominantly in the mononuclear cell–rich infiltrates of the deeper dermis and within neutrophils (**D**) (lysozyme), adjacent to the necrotic center in the upper dermis. Double immunolabeling was performed with CD68 (KP1), HLA-DR, and lysozyme staining in red, and *O. tsutsugamushi* in green. All biopsies were taken at day 7 postinoculation. (A) NHP ID 6856. (B) NHP ID5234. (C) ID5234. (D) ID5234. Images were acquired by confocal laser-scanning microscope (Zeiss LSM 700) using AxioVision LE Software v4.8.1 (Carl Zeiss Imaging Solutions GmbH, Germany) and optimized with Photoshop CS3 extended, version 10.0. Scale bars, 20 μm (A and C); 10 μm (B and D). Original magnification ×400 (A and C), ×1000 (B and D).

Eschar biopsies were collected from two macaques at two time points (days 2 and 7, biopsied macaques, *n* = 4) for double immunolabeling. The day 2 biopsies (ID5253 and ID8749) localized *O. tsutsugamushi* predominantly in the basal zones within sebaceous glands adjacent to hair follicles of the upper dermis (Fig. 4). Day 7 biopsies (ID5234 and ID6856) localized *O. tsutsugamushi* in the dermis, with mixed inflammatory subdermal perivascular infiltrates characterized by a large proportion of CD3^+^ T lymphocytes and APCs with high expression levels of MHC class II receptors (HLA-DR; Figs. 4 and 5). In the deeper dermis, the formation of lymphocyte follicles, with localization of *O. tsutsugamushi* at the peripheral border, was more prominent, representing a panniculitis. Colocalization studies revealed an association and intracellular location of *O. tsutsugamushi* with APCs, macrophages and neutrophils, staining positive for HLA-DR^+^, CD68^+^, and lysozyme^+^, respectively (Fig. 5).

Eschar biopsies were quartered and one specimen underwent qPCR assessment for *O. tsutsugamushi* and a Rhesus housekeeping gene to determine GEs (*O. tsutsugamushi* genomic equivalent [OTGE]). The eschar bacterial loads at day 2 for ID8749 and ID5253 were 413 and 1106 OTGEs/10^6^ cells, respectively. At day 7, the eschar bacterial load for ID6856 was negative and for ID5234 was 629 OTGEs/10^3^ cells, reflecting a strong decline of *O. tsutsugamushi* load in eschars over time.

#### Adaptive immune responses.

**FIGURE 6.**
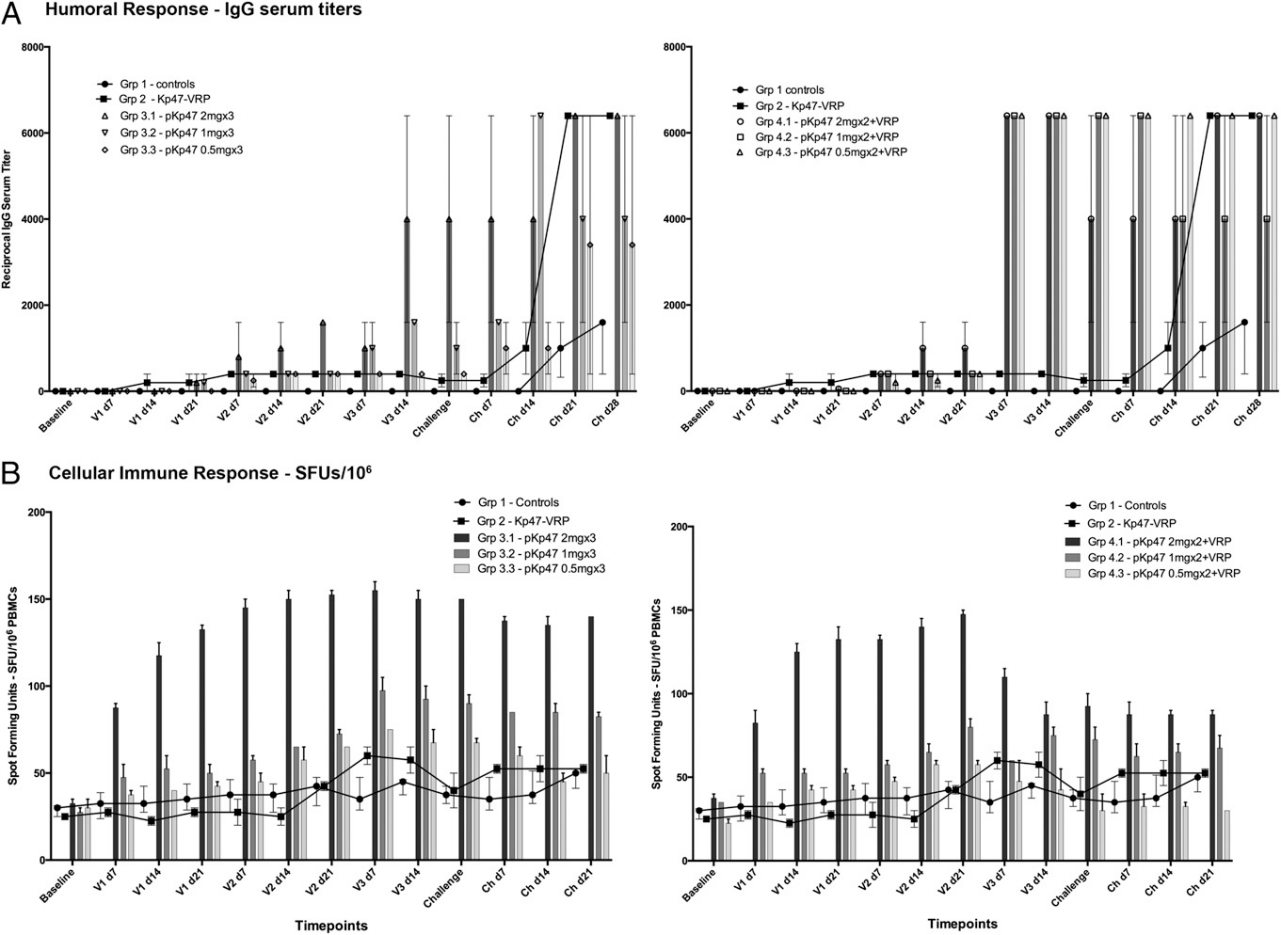
Humoral and cell-mediated immune responses of macaques. (**A**) Vaccination elicits an immune response reflected by earlier and higher levels of IgG in pKarp47 vaccine regimens than in Kp47/47-VRP vaccine regimens. The elicited humoral immune response dynamics differed between the cynomolgus vaccine groups; the triple-homologous plasmid pKarp47 vaccine regimen (group 3) was slow to produce high IgG titers (*left panel*), whereas the heterologous plasmid pKarp47 vaccine regimen using a boost with Kp47/47-VRP (group 4) induced earlier and higher levels of IgG (*right panel*). The Kp47/47-VRP vaccine regimen achieved the lowest IgG titers, which, however, increased rapidly upon natural challenge with *O. tsutsugamushi*. (**B**) Vaccination using a plasmid pKarp47-based vaccine regimen (group 3) elicits higher and more sustained cell-mediated immune responses than other vaccine candidates in this study. Time-course diagram of the cellular immune response after ex vivo stimulation of PBMCs depicting that vaccinees receiving the triple-homologous plasmid pKarp47 vaccine regimen (group 3) achieved the most pronounced cell-mediated immune response of all vaccine groups in this study, with sustained high numbers of IFN-γ–producing PBMCs, whereas macaques in the heterologous prime-boost regimen (group 4) showed a marked decrease of SFUs after the third vaccine, a boost with Kp47/47-VRP, was applied (B). Data are actual values. The number of IFN-γ–producing PBMCs is defined as SFUs per 1 million PBMCs. Time points include baseline, first, second, and third vaccine (V1, V2, and V3), respectively, and challenge (Ch), followed by days postvaccine or postchallenge (p.ex. V2 d14 corresponds to 14 d after second vaccination).

An *O. tsutsugamushi*–specific humoral response with increase of both IgM and IgG Ab levels occurred in cynomolgus macaques 1–3 wk after ID inoculation with *O. tsutsugamushi* Karp. In parallel, an increase in Kp r47b-specific IFN-γ–producing PBMCs, from an average of ∼30 to ∼45 spot-forming units (SFU)/10^6^ cells, at 3 wk after ID challenge with *O. tsutsugamushi* Karp was observed (Fig. 6).

#### Necropsy.

All macaques with autopsy immunohistochemical evidence of *O. tsutsugamushi* were control animals, that is, ID8749, ID6741 (PBS controls), ID5671 (pVR1012 control), ID5199 (pGM-CSF control), and ID6856 (mSEB-VRP control).

##### Histopathology.

Histopathological evaluations of the examined tissues were generally characterized by perivascular infiltrates consisting of histiocytic and lymphoplasmacytic inflammation with occasional mild hemorrhage. At necropsy, the eschar biopsy showed a histiocytic and lymphoplasmacytic cellulitis within the subcuticular tissues, frequently with evidence of mild chronic perivascular hemorrhage, erythrophagocytosis, and both extracellular and intrahistiocytic hemosiderosis. Rarely, admixed with the hemorrhage were mild-to-moderate amounts of necrotic debris and infiltrates of neutrophils. Regional lymph nodes frequently contained a moderate-to-marked sinus histiocytosis with mild-to-moderate lymphoid hyperplasia and frequent histiocytic intracytoplasmic cellular debris. In one animal, the splenic periarteriolar lymphoid sheaths were surrounded and mildly infiltrated by aggregates of histiocytes. Additional findings included minimal-to-mild tubular epithelial degeneration and necrosis. A focal and minimal granulomatous nephritis, concentrated on iridescent, retractile crystals, was seen in one animal but was interpreted to be a background aging lesion. Other incidental background lesions variably seen in these animals included cardiomyocyte megalokaryosis, hepatic lipidosis and glycogenosis, chronic lymphoplasmacytic salivary gland adenitis, and lymph node/pulmonary anthracosilicosis. No evidence of widespread or severe hepatic, pulmonary, cardiac, or cerebral inflammation was noted in any animal in autopsies after convalescence from scrub typhus, specifically no inflammatory “typhus nodules” as previously described in autopsy case reports from fatal human cases of acute scrub typhus (35).

##### qPCR of tissues.

The Otsu22 qPCR assay was performed on a series of autopsy tissues (*n* = 15) from CNS, skin, lymph nodes, lung, liver, spleen, kidney, and heart for the six control macaques. At 6 wk postinoculation, only one sample was found positive by qPCR, the inguinal lymph node of ID6741-PBS control (ipsilateral to eschar), with a density ratio of 40 *O. tsutsugamushi* GEs per 10^6^ cells. All CSF samples were negative.

##### Immunophenotyping of infected cells.

Cellular phenotyping of infected cells after euthanasia and completion of a natural disease course of scrub typhus revealed HLA-DR^+^ APCs and CD68^+^ (KP1) macrophages as the predominant cell phenotypes associated with intracellular *O. tsutsugamushi*. Organs with immunohistochemical evidence of *O. tsutsugamushi* at this time point were spleen (ID5671, ID6741), liver (ID5199), lymph node ipsilateral to eschar (ID6741, ID8749, and ID6856) and lymph node contralateral to eschar (ID8749).

### Immunogenicity and efficacy related to vaccination

#### Inoculation responses of vaccinated macaques.

The inoculation lesions in the vaccinated macaque groups varied from pronounced eschars to complete absence of lesion formation. Vaccinees receiving the Kp47/47-VRP vaccine (group 2) as a homologous triple-boost scheme (group 2) developed the largest and most pronounced lesions with necrotic centers, characterized by an early onset of edema and erythema (Fig. 1 and Table I). Four of five (80%) vaccinees receiving the triple pKarp47(×3) prime-boost scheme with three identical vaccinations (group 3) did not develop eschars. Although the eschar observed in group 3 (ID5732) had similar Draize and eschar scores as controls, its development was delayed by 4 d compared with controls. Another papulonodular lesion was observed in group 3, which did not classify as an eschar, because of absence of ulceration, necrosis, or crust formation, despite livid discoloration. Group 3 vaccinees had a significant lower frequency of eschar formation as compared with controls (*p* < 0.01, Pearson correlation). The macaques that did not receive this vaccine in this study had an 18-fold greater risk for development of an eschar (*p* = 0.034; OR [95% CI] 18 [1.24–261]).

Two of five macaques (40%) in group 4, pKarp47(×2) + Kp47/47-VRP(×1), did not develop eschars. The three with eschars were comparable in Draize and eschar scores to controls; however, two of three developed with a delayed onset of 4 d (Fig. 1 and Table I).

#### Lymphadenopathy of vaccinated macaques.

All challenged macaques developed inguinal lymphadenopathy ipsilateral to the inoculation site followed by subsequent generalized lymphadenopathy. Regional lymph-node enlargement was first observed at day 5 in all vaccine groups. Group 2, 3, and 4 macaques developed regional lymphadenopathy, at a median (range) of 5 (all were 5), 7 (5–9), and 5 (all were 5) days, respectively (Table I). The median (range) time to development of generalized lymphadenopathy was 12 (7–16), 16 (7–28), and 11 (7–19) days for group 2, 3, and 4 macaques, respectively. Although regional lymphadenopathy occurred almost simultaneously in all macaques, the appearance of generalized lymph-node enlargement was staggered by vaccine group, with the greatest delay in group 3; however, this was not significantly longer than controls in any vaccine group.

#### Temperature of vaccinated macaques.

All macaques demonstrated similar temperature curves, however, with differences of temperature peak onset, extent, and duration per vaccine group (Fig. 3). Vaccinees in group 2 with Kp47/47-VRP(×3) had an earlier onset in temperature by 3 d compared with controls, with significantly earlier and higher temperatures at day 9 postchallenge (*p* < 0.0001). Homologous prime-boost vaccinees in group 3, with pKarp47(×3), demonstrated a delayed fever curve with maximum temperature of 39.0°C at day 16 (peaking 2 d after controls), and a right shift with significantly lower temperatures at days 12 and 14, compared with controls (*p* = 0.0001). The heterologous prime-boost vaccinees in group 4, with pKarp47(×2) + Kp47/47-VRP(×1), followed a similar fever profile as the control group with no significant differences in core temperature.

Transformation of fever data into AUCs for comparisons revealed no significant differences between vaccine groups, although for all macaques the temperature AUC correlated with the density of bacteremia, suggesting that higher fever correlates with higher bacterial loads (*p* = 0.009, Spearman’s rank correlation).

#### Bacteremia.

The period of bacteremia (in days), the onset of bacteremia (first qPCR positivity according to days postinoculation), and the extent of bacteremia quantified as OTGE/10 μl whole blood) were observed among the three groups of vaccinated macaques in this study (Table II).

##### Sterile immunity.

All control macaques demonstrated bacteremia, including those that underwent biopsy at days 2 and 7. The absence of *O. tsutsugamushi* DNA in blood for 28 d after challenge was defined as sterile immunity. This was achieved in 3/12 (25%) of all vaccinated macaques, specifically in 0/2 for group 2, in 2/5 (40%) for group 3, and in 1/5 (20%) for group 4 macaques. A third macaque in group 3 (ID5253D) had no evidence of bacteremia; however, this animal had a biopsy of the injection site 2 d after inoculation, which could have affected this outcome through an additional reduction of bacterial inoculum via excision.

##### Onset of bacteremia.

The time to onset of bacteremia (TTB) was shortest in the control group, with a median (IQR) of 9 (8–10.5) d postinoculation, followed by 10.5 (9–12) d in group 2, and was prolonged to 14 (12–19) and 13 (12–14) d postinoculation in groups 3 and 4, respectively. TTB for vaccine groups 3 and 4, but not for group 2, were significantly longer than for controls (*p* = 0.048 and 0.024, respectively).

##### Duration of bacteremia.

The median (IQR) duration of bacteremia for control macaques was 12 (10.5–14) d, after excluding biopsied macaques. Macaques in group 3 showed the shortest period of bacteremia with a median of 5 (0–6) d, followed by 8 (8–12) d in group 4 and 9 (5–13) d in group 2. The duration of bacteremia ranged from 5 to 15 d across all groups. Group 3, but not groups 2 and 4, vaccinees had the shortest duration of bacteremia, significantly shorter than controls (*p* = 0.014).

##### Quantitation of bacteremia, estimated biomass.

Bacterial loads expressed as median GEs (IQR) and AUC calculations served as surrogate estimates for the circulating biomass and were calculated per group. Controls had a median of 7 GEs (6.5–10.5) and an AUC of 297 (150–1140), whereas group 2 macaques had 13 GEs (8–18) and an AUC of 1104 (61–2148). Group 3 macaques had the lowest bacterial loads corresponding to 5 GEs (4–9) and lowest AUC of 22 (0–28), followed by group 4 with 8 GEs (2–11) and AUC of 142 (9–277), respectively. Comparisons of bacterial control upon challenge expressed as AUC differed significantly between controls and vaccines in group 3 (*p* = 0.014, Kruskal–Wallis); the other comparisons did not reach significance.

#### Bacteremia indices per vaccine group.

Vaccinees with the homologous prime-boost protein-based Kp47/47-VRP(×3) vaccine candidate (group 2) showed no significant reduction in the duration or onset of bacteremia when compared with the control group, and actually had increased bacterial loads and AUCs, respectively.

Vaccinees with the homologous prime boost with the pKarp47 vaccine candidate (group 3) had a significantly shorter bacteremia duration (*p* = 0.014), a delayed onset of bacteremia (*p* = 0.048), and the lowest bacterial loads or AUCs in the study (*p* = 0.014).

Vaccinees with the heterologous prime-boost vaccine group 4, pKarp47 + Kp47/47-VRP, had a delayed onset of bacteremia (*p* = 0.024), but did not have a significant reduction of bacteremia duration (*p* = 0.14), or lower bacterial loads or AUCs (*p* = 0.33) when compared with controls.

#### Safety of vaccines (biochemistry and hematology).

A large panel of biochemical and hematological markers (*n* = 26) was measured at 14 time points throughout the study (baseline, 3 time points per vaccination, and 4 during challenge), and results did not reveal any abnormalities attributable to a vaccine (Supplemental Material).

The following markers were associated with changes between vaccine groups at three or more time points (*p* < 0.05 in either Kruskal–Wallis or rank sum tests) leading up to challenge.

##### Total protein.

At weeks 3, 5, 6, 8, and 9, all members in vaccine group 3 demonstrated significantly lower total protein levels compared with other groups. At weeks 3, 5, 7, and 8, vaccine group 4 macaques demonstrated significantly lower total protein level values compared with other groups (lowest value = 44 g/L).

##### Liver transaminases.

At weeks 2, 6, 8, and 12, ALT levels of group 3 macaques were significantly increased compared with other groups; however, the change was minimal because values did not reach double-baseline values (maximum value = 74 U/l).

#### Effect of vaccines on biochemistry serum markers.

Baseline biochemistry values were compared with measurements during live challenge (at days 7, 14, 21, and 28) and compared between control and vaccine groups. No significant differences in change of hematological markers related to vaccine groups were observed, but the following biochemical markers were significantly altered in the vaccine groups as compared with controls (rank sum test): group 2—increased globulins (*p* = 0.032, day 14), decreased albumin/globulin ratio (*p* = 0.045, day 14), and decreased alanine aminotransferase levels (*p* = 0.043, day 21), when compared with controls; group 3—decreased total protein (*p* = 0.032, day 14; *p* = 0.023, day 28), decreased globulins (*p* = 0.006, day 28), increased albumin/globulin ratio (*p* = 0.006, day 28), decreased triglycerides (*p* = 0.025, day 14; *p* = 0.037, day 28), increased cholesterol (*p* = 0.009, day 14), decreased aspartate aminotransferase (*p* = 0.036, day 21), decreased alkaline phosphatase (*p* = 0.037, day 21), when compared with controls; and group 4—increased albumin/globulin ratio (*p* = 0.008, day 28), decreased aspartate aminotransferase (*p* = 0.01, day 7, *p* = 0.053, day 14), and decreased alanine aminotransferase (*p* = 0.029, day 7), when compared with controls.

#### Immune responses to vaccination.

##### ELISA assays.

Ag-specific IgM and IgG titers were detected in the sera of macaques immunized with Kp47/47-VRP (group 2), pKarp47(×3) (group 3), and pKarp47(×2) + Kp47/47-VRP(×1) (group 4). IgM Abs reacting to the Kp r47b ELISA Ag preparation were first detected among macaques immunized with pKarp47 3 wk after the first vaccination in 3 of 12 individuals, 1 wk after the second vaccination in 10 of 12 individuals, and in all 12 macaques 2 wk after the second vaccination (data not shown). One week after the 2 vaccinations with pKarp47 and 1 vaccination of the Kp47/47-VRP, all 6 monkey Ab titers increased sharply to ≥6400, whereas in the 6 monkeys that received 3 doses of pKarp47, Ag-specific Ab titer increased only 4-fold or not at all.

Ag-specific IgGs induced by vaccination with Kp47/47-VRP (group 2) were detected in one of two macaques 2 wk after the initial vaccination and in both macaques after the second vaccination. IgG Abs to Kp47/47-VRP increased to detectable levels quicker than to pKarp47(×3); however, the titer to Kp47/47-VRP never increased to >1:400, whereas the titer of IgG to pKarp47(×2) + kp47/47-VRP(×1) increased to 1:6400 upon application of the third vaccine (Fig. 6A). All Abs to the immunogens were of subclass IgG1 and not IgG2, IgG3, or IgG4 (data not shown). Unvaccinated control monkeys, as expected, were consistently negative for Abs to Kpr47b during the same period.

##### ELISpot assays.

**FIGURE 7.**
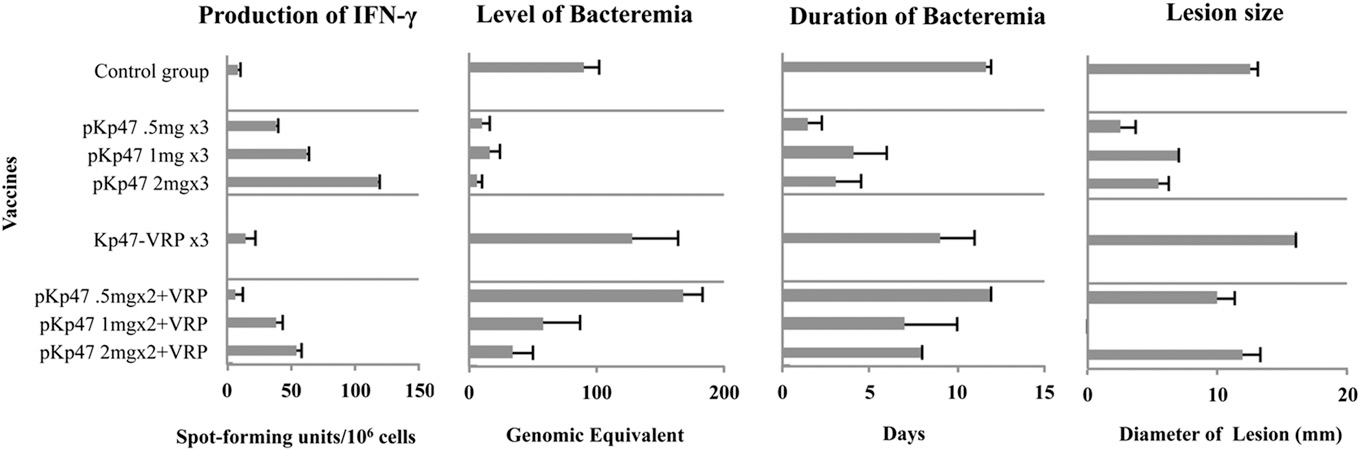
Relationship among IFN-γ–producing PBMCs, level and duration of bacteremia, and diameter of lesion postinoculation between vaccine groups. The left graph summarizes the number of Ag-specific, IFN-γ–producing PBMCs collected at 3 wk after third vaccination, as estimate of cellular immunity available before challenge with *O. tsutsugamushi*. Vaccinees with the pKarp47 ×3 vaccine (group 3) had the highest IFN-γ–producing PBMCs, the lowest bacteremia, and the shortest bacteremic duration. The middle graphs depict the median levels of bacteremia and duration of bacteremic dissemination phase during acute disease, and the right graph depicts the diameter of the eschar lesion at day 12 per group (and dosage regimens).

In group 1, the unvaccinated control macaques did not exhibit an increase in the number of Ag-specific, IFN-γ–secreting cells until day 21 after ID challenge with *O. tsutsugamushi* Karp. In group 2, homologous triple vaccination with Kp47/47-VRP induced few IFN-γ–producing cells, with an increase of SFUs in the ELISpot assay visible only after the third vaccination (Fig. 6B). In group 3, PBMCs from animals immunized with pKarp47(×3) produced Ag-specific IFN-γ in a dose-dependent manner. These vaccines had a significantly higher median number of IFN-γ–producing cells than controls (*p* = 0.043, Mann–Whitney *U* test). The macaques receiving the highest dose of pKarp47(×3) in group 3 showed early and strong increases in Ag-specific IFN-γ productions in the ELISpot assays (Fig. 7). The same was observed for group 4 macaques after the first two vaccines were applied (which were pKarp47), but the number of IFN-γ–secreting cells declined after administration of the boost vaccination with Kp47/47-VRP (Fig. 6B). Levels of IL-13 in vaccinated animals did not reach levels above control animals (data not shown).

##### Sterile immunity.

In this study, sterile immunity was defined as lack of evidence for bacteremia during the following 28 d after live challenge (= end-of-study time point) in the presence of a cell-mediated immune response. Of the vaccinated macaques, 3/12 (25%) achieved sterile immunity and 6/12 (50%) did not develop lesions that classified as eschars. The macaques with sterile immunity did not develop an eschar; one had a small papular lesion at the inoculation site, similar to previous human subjects with pre-existing immunity (36). Although a fourth macaque had sterile immunity, it remained unclear whether this was attributable to vaccine or skin biopsy taken at day 2.

**FIGURE 8.**
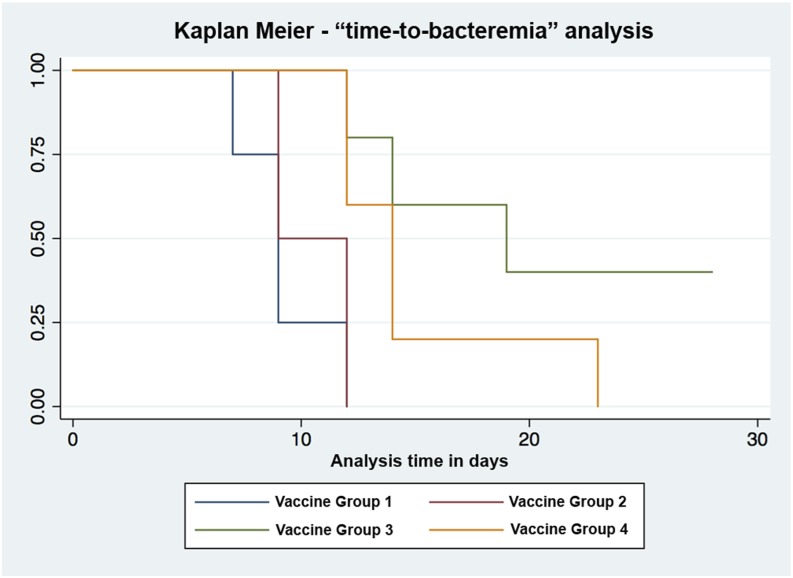
Kaplan–Meier curves based on TTB end points demonstrate sterile immunity in group 3 vaccinees (homogenous triple-prime-boost pKarp47 regimen). The duration from ID inoculation of *O. tsutsugamushi* (challenge time point) until onset of bacteremia was termed TTB, and the onset of bacteremia served as the end point for a Kaplan–Meier survival analysis. A significant reduction of TTB was associated with plasmid DNA vaccine regimens of groups 3 (hazard ratio 0.073; *p* = 0.006) and 4 (hazard ratio 0.172; *p* = 0.032). The benefit in reducing bacteremia attributable to the homologous triple-boost regimen pKarp47(×3) was 2.35-fold higher than for the heterologous prime-boost pKarp47(×2)+Kp47/47-VRP vaccine candidate.

The pKarp47(×3) vaccine candidate (group 3) provided the best immune protection, with 2/5 (40%) macaques representing 2 different doses (2 and 0.5 mg/individual, respectively) demonstrating sterile immunity (Fig. 8). Vaccine candidate pKarp47(×2) + Kp47/47-VRP(×1) also achieved sterile immunity in one macaque; although the results were not as pronounced as in vaccine group 3, there was an increased time to detectable bacteremia in vaccinees, with reduction of bacteremic duration and bacterial loads (AUC) when compared with controls. The nonreplicating virus-based candidate Kp47/47-VRP showed the least benefits with no sterile immunity. Although a reduction of the bacteremic period from 12 to 9 d was observed, this virus-based vaccine formulation resulted in higher bacterial loads and AUCs than the other candidates.

#### Relationship of IFN-γ production and protection from infection with *O. tsutsugamushi*.

In all macaques an increase of IFN-γ–producing cells was associated with a smaller bacteremia-AUC relating to a reduced bacterial biomass (*p* = 0.014, Spearman’s rank correlation), a prolonged TTB, relating to a delayed onset of bacterial dissemination (*p* = 0.019, Spearman’s rank correlation), and a shorter duration of bacteremia relating to an improved control of bacteremic dissemination (*p* = 0.026, Spearman’s rank correlation). Although temperature-AUCs correlated to bacteremia-AUC (*p* = 0.009, Spearman’s rank correlation), they did not inversely correlate with increasing IFN-γ–producing cells, as expected (*p* = 0.09, Spearman’s rank correlation). This could be attributable to sample size, and thus insufficient power or alternative factors influencing the temperature curves.

Group 3 vaccinees achieved the most pronounced cell-mediated immune response in this study, demonstrating a sustained high number of IFN-γ–producing PBMCs after homologous triple-boost pKarp47(×3) vaccinations (*p* = 0.043, Mann–Whitney *U* test). This group also demonstrated the highest number of macaques with sterile immunity (in 40% of vaccinees), a significantly lower frequency of eschar formation compared with controls (80% without eschar formation; *p* < 0.01, Pearson correlation), the strongest decrease in diameter of inoculum lesions (if present), the shortest duration of bacteremia, the longest duration until bacteremia onset, the lowest bacterial loads as expressed by AUC, as well as the highest reduction of liver transaminases (Figs. 1, 3, and 7 and Table I). These results are suggestive of a possible relationship between IFN-γ levels induced by vaccination and immune protection.

## Discussion

This study characterized a cynomolgus macaque ID challenge model of human scrub typhus, and evaluated the safety, immunogenicity, and efficacy of two candidate vaccines: the pKarp47 and Kp47/47-VRP vaccines, each applied via homologous triple-boost regimens and a heterologous prime-boost regimen.

This study demonstrated that a vaccine-induced response is capable of conferring sterile immunity against high-dose ID challenge of *O. tsutsugamushi* in an NHP model, hitherto only seen in homologous rechallenge after natural infection with the same strain of *O. tsutsugamushi* (36). This study validated the ID challenge cynomolgus macaque model on the basis of its accordance with human scrub typhus disease and confirms similar bacterial cellular tropism in the eschar (21). The model also provided insight into *O. tsutsugamushi* dissemination dynamics, under the influence of various vaccine-induced levels of cell-mediated and humoral immune responses, demonstrated the importance of bacteremia indices for evaluation of the immune response(s), and importantly, to our knowledge, has enabled the determination of the first phenotypic correlates of immune protection in scrub typhus.

### Cynomolgus macaques replicate mild disease features of human scrub typhus

Human scrub typhus is characterized by fever, nonspecific symptoms, an early bacteremic period, possible eschar formation, and a strong association with regional lymphadenopathy after ID inoculation of *O. tsutsugamushi* via *Leptotrombidium* chigger bite (21, 37, 38). This study confirmed that clinical features of human scrub typhus are similar in cynomolgus macaques, in that ID needle inoculation of *O. tsutsugamushi* results in a febrile illness with inoculation eschar, local and generalized lymphadenopathy, a phase of bacteremia, and development of humoral and cellular immune responses (19, 20, 39). No generalized symptoms like malaise, anorexia, and weakness, or complications reflecting severe disease in humans (meningoencephalitis and pneumonitis) were apparent in cynomolgus macaques during the course of this study, but biochemical and hematological changes mimicked human disease. Although *O. tsutsugamushi* Gilliam strain previously demonstrated high pathogenicity in cynomolgus macaques (26), this study evaluated the Karp strain, a human lethal strain, because it is the most representative of the human-pathogenic strains found across Asia (40, 41). Using an inoculum of 10^6^ MuLD_50_ resulted in a relatively low disease severity, which could be related to either the inoculum dosage, the strain used, or the macaques themselves.

### Clinical features of scrub typhus in cynomolgus macaques

The major strength of this macaque model is the development of classic eschar lesions with explicit lymphadenopathy (Figs. 1 and 2) similar to humans, and no other animal model in scrub typhus has reliably reproduced this distinctive inoculation lesion upon ID inoculation to date (11, 18, 20). The ID route has previously been shown to induce the same disease course and dynamics in humans as in naturally mite-transmitted scrub typhus (36, 42), and this study confirms the same findings of this inoculation approach in an NHP model.

All control macaques developed fever on the eighth to tenth days, comparable with human volunteers (36, 43) and silvered leaf macaques (26) after ID inoculation of *O. tsutsugamushi* Gilliam strain. Only macaques that developed bacteremia had elevated temperatures, and temperature curves followed the same dynamics as bacteremia as measured by qPCR in blood (Fig. 3); in the first week after inoculation, a temperature decline was followed by a peak at 2 wk to normalize in the fourth week. The significance of this initial temperature decline remains to be elucidated, although a role of cytokines in the early immune response seems probable. Regional lymphadenopathy was established in all macaques during this temperature trough, before establishment of blood PCR-positivity, eschar formation, and fever. The eschars developed to maximum scores at days 14–16, coinciding with peak temperature and bacteremia, followed by establishment of generalized lymphadenopathy, as previously observed in humans (36, 42).

### Effects of vaccination on clinical features

All control macaques developed eschars, but the local responses to challenge varied from pronounced eschars to complete absence in the vaccine groups, for example, the incidence of eschar formation in group 3 vaccinees was significantly lower than in controls (*p* < 0.001). We observed that macaques without eschars demonstrated either sterile immunity or low bacterial loads with delayed onset (Table II), suggestive of a relationship between the ability to control bacteremia and eschar formation. Because previous human challenge studies from the 1950s suggest a relationship between eschar formation and immune protection (36, 42, 44), we evaluated these parameters in this animal model.

Macaques with high numbers of IFN-γ–producing cells generally had lower bacterial biomass (*p* = 0.014), with shorter bacteremia (*p* = 0.026) and delayed onset of bacteremia (*p* = 0.019), suggestive of cell-mediated immunity contributing to the control of bacteremia (Fig. 7). Vaccinated macaques with better control of bacteremia also had the lowest fever AUCs, and macaques achieving sterile immunity had no febrile episodes (Table III). Observations in the 1970s suggested a relationship of bacteremia to fever, and our cynomolgus model time-course data have now demonstrated that fever correlates with the extent of bacterial biomass (*p* = 0.009, Spearman’s rank correlation) (26, 45). This finding is important and has clinical implications, because every febrile patient with clinical suspicion of scrub typhus could potentially be diagnosed with a positive qPCR result, according to the detection limits of the assay.

**Table III.** Phenotypic correlates associated with immune protection

Sterile immunity	Absence of bacteremia in the presence of cellular and humoral immunity
Fever	Delayed onset, with right shift or protracted fever curves, and reduction of fever AUC
Eschar	Absence of eschars, delayed time to onset, decreased size and severity score
Bacteremia	Reduction of duration, TTB, and total bacterial biomass (AUC)
Biochemistry markers	Total protein, albumin, albumin/globulin ratio, and liver transaminases

These phenotypic markers were significantly associated with immune protection associated with high frequencies of IFN-γ–secreting, Ag-specific T cell responses in cynomolgus macaques.

The absence of an eschar correlated negatively with the extent of bacterial biomass (*p* = 0.016), the bacteremia duration (*p* = 0.003), and time to bacteremia (*p* = 0.003), which suggests that an early protective immune response, as evidenced by successful control of bacteremic dissemination, prevents local eschar formation. However, in this study, high levels of IFN-γ–producing cells did not correlate significantly with the presence of an eschar. The limited sample size of this study did not provide sufficient power to determine whether this association is true or whether additional immune mediators affect eschar formation.

The delayed onset of fever and the slower development of the cutaneous lesions observed in vaccinated macaques with proven cell-mediated immune responses resemble previous observations in human volunteers with pre-existing immunity and reinoculation in a field study in 1950 (36). We previously showed that a stepwise reduction in bacterial inoculation loads (10^6^ to 10^0^ MuLD_50_) translated into a stepwise delayed onset of bacteremia (19). In this study, the inoculation load at challenge (10^6^ MuLD_50_) was the same for all macaques, but immune protection varied with vaccine formulation as measured by IFN-γ–producing cells. The association of efficient control of bacteremia with vaccine-associated immunity reflects partial immune protection and that the bacteremia indices, duration, time to onset, and bacterial biomass (AUC), are representative end points for evaluating immune responses in scrub typhus.

### *O. tsutsugamushi* colocalize with APCs and neutrophils in eschar biopsies

The localization of *O. tsutsugamushi* in eschar skin biopsies at day 2 was predominantly within and around sebaceous glands and hair follicles, whereas at day 7 generally fewer bacteria were seen, typically located within perivascular mononuclear cell infiltrates in the dermis (Figs. 4 and 5). The day 7 findings showed high numbers of neutrophils along the perimeter of the necrotic zone, *O. tsutsugamushi*–infected APCs in the mononuclear cell–rich infiltrates of the deeper dermis, and macrophages and neutrophils in the upper dermis. This picture is identical to results previously described in human eschars, further supporting the usefulness of this macaque model (21). Immunohistochemical examination of autopsy tissue samples revealed persistence of *O. tsutsugamushi* at 30 d postinoculum, after completion of the natural disease course, in tissues of the reticuloendothelial system, with lymph nodes, spleen, liver, and eschar containing *O. tsutsugamushi*–infected APCs and macrophages. These cell phenotypes could play a role in the persistence of *O. tsutsugamushi* in long-term or latent infections, as previous discussions have highlighted (46, 47). The fact that bacterial persistence occurs in tissues of the lymphoreticular system at day 30, without significant host inflammatory response, supports the possibility that intracellular bacteria may contribute to a dormant/latent state of infection.

The two macaques that had eschar biopsies taken at day 2 demonstrated a decreased duration and delayed onset of bacteremia with lower bacterial loads (AUC) than other members of their vaccine group, with one macaque in group 3 achieving sterile immunity. The effects associated with these biopsies most likely reflect the reduced (excised) inoculum load, because macaques that underwent biopsy at a later stage demonstrated bacteremia indices similar to controls, suggestive of effective dissemination from the eschar site to draining lymph nodes at ∼7 d. These observations further support a relationship among inoculum load, eschar formation, and bacteremia, and raise the possibility that a vigorous immune reaction at the inoculation site, with rapid influx of neutrophils, mononuclear cells, and macrophages, reduces inoculum dissemination and consequently bacteremia (21). An immune surveillance mechanism capable of rapid bacterial detection in situ in the skin, mediated by skin-resident immune memory and/or effector cells, could induce rapid early immune activation in the regional lymph node, resulting in effective and rapid clearance of intracellularly infected cells arriving via the lymphatic vessels (48). These observations require confirmation via further functional immunological time-course studies to elucidate the mechanisms of immune protection.

### Biochemistry and hematology

Similar to previous observations in silvered leaf macaques, a reduction of WBCs, predominantly because of lymphopenia, and thrombocytopenia was observed during the period of bacteremia in controls during the natural disease course (26). Although elevation of liver enzymes is common in human scrub typhus, cynomolgus macaques did not develop significantly increased AST or alanine aminotransferase plasma levels from baseline during challenge. This is probably a reflection of the low disease severity attained in this study. A significant reduction of total protein and liver transaminases compared with controls in the bacteremic phase was observed for macaques receiving the pKarp47(×3) and pKarp47(×2) + Kp47/47-VRP(×1) vaccine candidates, which could be attributable to the vaccine-related effects on bacteremia, and thus reduction of disease severity. This study was not designed to evaluate disease severity parameters, and future investigations are planned to address these findings in more detail.

### DNA plasmid vaccines induce a protective immune response in cynomolgus macaques

Alphavirus replicon particle vectors using VEE induce robust cellular, humoral, and mucosal immune responses specific for the replicon-expressed Ag in a cynomolgus macaque model (49). DNA plasmid vaccines and replication-deficient viral vectors such as VEE encoding target Ags are current strategies to safely induce cellular immunity (28, 50, 51). The two vaccine formulations used in this study can be considered safe, and they were not linked to any clinical signs or symptoms, or biochemical or hematological markers suggestive of vaccine-related side effects during the period leading up to challenge.

The most desirable end point for a protective vaccine is long-lasting sterile immunity. We found evidence for sterile immunity in 3/12 (25%) macaques receiving a DNA plasmid-based vaccine regimen: group 3 (*n* = 2), group 4 (*n* = 1; Fig. 8), and none of the macaques attaining sterile immunity developed eschars. Further, all macaques without an inoculation eschar (*n* = 6/12, 50%) had either no evidence of bacteremia or delayed and extremely low bacteremia indices, supporting the relationship between these two immune phenotypic end points (Table II). Group 3 vaccinees, which had the lowest frequency of eschars (*p* < 0.01) of all groups, also responded with the highest numbers of IFN-γ–secreting cells, and strongest cell-mediated immune protection in this study (*p* = 0.043). Including all study participants, those macaques that did not receive the pKarp47(×3) vaccine were at an 18-fold greater risk for development of an eschar (*p* = 0.034, OR [95% CI], 18 [1.24–261]). It is apparent that the induction of a strong cell-mediated immune response reduces bacteremia and eschar development, and induces resistance to (re)infection in this model, which replicates previous findings in humans (36, 42).

An unexpected finding was that macaques receiving the VRP-based vaccine (Kp47/47-VRP, group 2) developed the most pronounced necrotic eschars, accompanied by poor control of bacteremia (Table II). Although IFN-γ–producing PBMC responses were weak and comparable with controls in this group, the low-level Ab titer achieved during the vaccination phase increased substantially upon live challenge suggestive of the natural challenge “boosting” the vaccine-induced immune response (Fig. 6). The results, albeit limited by small sample size, suggest that poor control of bacteremia could be attributable to a lack of cell-mediated immunity in the presence of high IgG Ab titers, and that a pre-existing humoral response (in the absence of a cellular response) could be associated with a more pronounced local reaction at the inoculation site, and possibly with more severe disease. A study in mice showed that immune serum may block *O. tsutsugamushi* surface proteins and inhibit a specific event required in the infection process (52, 53). These, however, were neutralizing Abs predominantly directed against the 56-kDa surface Ag (54). In this study, the effect of high anti–47-kDa Abs in the presence of low cell-mediated immunity appears to be inversely associated with the inhibition of eschar formation and control of bacteremia. At the time of this study, it was thought that a dose of 10^7^ FFU Kp47/47-VRP would be sufficient to induce protective immune responses, as compared with Marburg virus MBGV-VRP that induced complete protective responses at a dose of 10^7^ FFUs in cynomolgus macaques (55). Since our study was conducted, other studies have shown that a dose of 10^10^ FFUs Sudan virus SUDV-VRP was necessary to confer protection in cynomolgus macaques against an Ebola virus challenge (49). Consequently, our study might have benefited greatly from using a higher dose of Kp47/47-VRP.

### Homologous and heterologous DNA plasmid vaccine regimens elicit divergent cell-mediated responses

Time-course comparisons of IgG and cell-mediated immune responses achieved by the homologous pKarp47(×3) and heterologous pKarp47(×2) + Kp47/47-VRP(×1) vaccine regimens revealed that high levels of IgG titers paralleled with variable levels of IFN-γ–secreting PBMCs (Figs. 6 and 7). The establishment of an early and strong cell-mediated immunity was achieved in both groups after two vaccinations using the highest dose of pKarp47 (Fig. 6B), but a distinct divergence of SPUs in the ELISpot assays became eminent at the third vaccination: whereas the homologous triple-boost scheme increased and maintained a strong cellular response, this was not apparent in group 4, resulting in a bifurcation and reduction of IFN-γ–producing PBMCs upon boost with Kp47/47-VRP.

Further, although group 3 vaccinees gradually built up a humoral response in a dose-dependent manner with IgG titers rising earlier and stronger in the high-dosage regimens, the group 4 vaccinees demonstrated an early, pronounced increase in Ab titer upon the third Kp47/47-VRP boost vaccination (Fig. 6). Although the median time to onset of bacteremia was significantly prolonged for both vaccine groups compared with controls (*p* = 0.006 and *p* = 0.032 with hazard ratios of 0.073 and 0.172 for groups 3 and 4, respectively), the benefit in reducing the onset of bacteremia in group 3 was 2.35-fold higher. Previous vaccination strategies in malaria achieved 5- to 10-fold higher IFN-γ–secreting, Ag-specific T cell responses in humans than responses induced by a DNA vaccine or recombinant MVA vaccine alone, and produced similar results with partial protection against malaria manifesting as delayed parasitemia after sporozoite challenge (56). Newer strategies that have substituted the priming plasmid DNA with a specific attenuated recombinant fowlpox virus (FP9) vaccine in prime-boost regimens elicited improved complete and longer sterile protection associated with persisting memory T cell responses (57).

Historically, IFN-γ and a type 1 immune response have been associated with protection from *O. tsutsugamushi* and rickettsial infections in animal models (58–60), and strong IFN-γ responses to *O. tsutsugamushi* infection have been associated with acute scrub typhus in humans (61–63). Our study has identified immune phenotypic correlates of a protective IFN-γ–secreting PBMC response in a cynomolgus macaque model of scrub typhus. The homologous triple-prime-boost plasmid DNA vaccine pKarp47(×3) achieved the highest number of *O. tsutsugamushi*–specific, sustained IFN-γ–producing PBMCs, which was associated with sterile immunity, the lowest frequency of eschar formation, the shortest duration of bacteremia, the longest duration until onset of bacteremia, the lowest bacterial biomass, as well as reduction of liver transaminases (Table III). Although the pKarp47(×3) vaccine induced protective immunity against scrub typhus after ID needle challenge in our NHP model, further characterization of the elicited immune responses and evaluation of further vaccine candidates are required. The current focus on protein Ags recognized by sera obtained from immunized animals and infected humans (64, 65) needs to be expanded to a broader genomewide approach for improving potential diagnostic targets and vaccine design. The use of a well-characterized NHP model is a prerequisite for this work. The results of this study support the usefulness of cynomolgus macaques, because the ID challenge route induces an infection that closely resembles clinical human disease, and enables further investigation of the mechanisms and phenotypic correlates of vaccine-induced immune responses.

## Supplementary Material

Data Supplement
